# Chitosan–Gelatin Films: Plasticizers/Nanofillers Affect Chain Interactions and Material Properties in Different Ways

**DOI:** 10.3390/polym14183797

**Published:** 2022-09-11

**Authors:** Qingfei Duan, Ying Chen, Long Yu, Fengwei Xie

**Affiliations:** 1 College of Food Science and Engineering, South China University of Technology, Guangzhou 510640, China; 2 School of Food Science and Engineering, Yangzhou University, Yangzhou 225127, China; 3 School of Engineering, Newcastle University, Newcastle upon Tyne NE1 7RU, UK

**Keywords:** chitosan, gelatin, biopolymer nanocomposites, biopolymer plasticization, glycerol, isosorbide

## Abstract

Biopolymers, which are biodegradable and inherently functional, have high potential for specialized applications (e.g., disposable and transient systems and biomedical treatment). For this, it is important to create composite materials with precisely defined chain interactions and tailored properties. This work shows that for a chitosan–gelatin material, both glycerol and isosorbide are effective plasticizers, but isosorbide could additionally disrupt the polyelectrolyte complexation (PEC) between the two biopolymers, which greatly impacts the glass transition temperature (*T*_g_), mechanical properties, and water absorption. While glycerol-plasticized samples without nanofiller or with graphene oxide (GO) showed minimal water uptake, the addition of isosorbide and/or montmorillonite (MMT) made the materials hydrolytically unstable, likely due to disrupted PEC. However, these samples showed an opposite trend in surface hydrophilicity, which means surface chemistry is controlled differently from chain structure. This work highlights different mechanisms that control the different properties of dual-biopolymer systems and provides an updated definition of biopolymer plasticization, and thus could provide important knowledge for the future design of biopolymer composite materials with tailored surface hydrophilicity, overall hygroscopicity, and mechanical properties that meet specific application needs.

## 1. Introduction

Nature biopolymers such as chitin/chitosan and gelatin are renewable, biodegradable, non-toxic, biocompatible, and inherently functional, and thus have aroused much attention for developing materials for disposal and transient systems and for biomedical treatment [1,2]. Chitosan is an FDA-approved polysaccharide composed of randomly distributed β-(1-4)-linked d-glucosamine (deacetylated) and *N*-acetyl-d-glucosamine (acetylated) units. Chitosan has inherent antimicrobial activity and excellent complex-forming ability, which can be mainly attributed to its availability of free —NH_2_ groups protonated and positively charged in acid conditions [3,4,5]. Gelatin, which is a soluble protein obtained from irreversible partial hydrolysis of collagen and has a triple-helical structure and a hydrophilic and ionic character, is becoming particularly attractive in the field of controlled release of drugs, tissue engineering, and biodegradable active food packaging [2,6,7,8]. Combining different biopolymers to create composites is an important means to achieve enhanced properties and/or functionality, which can better meet application needs. Due to the polyelectrolyte and polyampholyte nature of chitosan and gelatin, respectively, they exhibit distinct properties in ionic/aqueous solutions and can form polyelectrolyte complexation (PEC) at a pH above the isoelectric point (pI) of gelatin and lower than the pI of chitosan [9,10,11]. Polyelectrolyte complexed biopolymer materials have been proved to have superior properties that single biopolymers cannot achieve, such as hydrolytic stability [12], barrier properties [13,14], mechanical properties [9,15], controlled-release ability [16,17], and cell adhesiveness to some extent [18]. Our previous study indicated that a 1:1 (*w*/*w*) chitosan–gelatin material had better mechanical properties and lower hygroscopicity than materials that are mainly based on chitosan or gelatin [19].

To improve the structural and functional properties (such as mechanical properties and moisture sensitivity) of biopolymers, nanomaterials have attracted wide attention as fillers due to their excellent physical and chemical properties (e.g., excellent mechanical properties, large surface area, and special functional groups) [20,21,22]. In this regard, 2D nanoparticles such as graphene oxide (GO) and montmorillonite (MMT) are especially interesting. Due to the presence of oxygen-containing polar groups (e.g., carboxylic acid and phenolic hydroxyl groups), GO is hydrophilic and compatible with biopolymers [23,24]. Moreover, the surfaces of GO sheets are highly negatively charged when dispersed in water, as a result of the ionization of carboxylic acid and phenolic hydroxyl groups on the GO sheets [23,25]. MMT clay is chemically represented as M*_x_*(Al_4–*x*_Mg*_x_*)Si_8_O_20_(OH)_4_, where M represents a monovalent cation and *x* the degree of isomorphous substitution, which can range between 0.5 and 1.3 [26]. Due to the presence of inorganic cations between the interlayer spaces (galleries), natural MMT is hydrophilic and thus miscible with hydrophilic polymers. Previous studies have shown that GO or MMT can effectively modify the properties of chitosan and gelatin materials. For example, it was reported that with the incorporation of 1 wt% GO, the Young’s modulus of a chitosan film was improved by about 64%, and the tensile strength increased by about 122% [23]. Wang et al. [27] reported that loading of up to 10 wt% MMT clay enhanced the thermal stability and improved the elastic modulus and hardness of a chitosan matrix. In addition, with the addition of MMT, a gelatin scaffold showed decreased swelling ratio and biodegradation rate and strengthened mechanical behavior [28]. Despite these prior attempts, there have been limited reports on chitosan–gelatin composites filled with GO or MMT. Moreover, there have been limited studies on chitosan-based nanocomposites prepared by high-viscosity thermomechanical processing [24,29], which is advantageous in creating bulk polyelectrolyte complexes (whereas in solution conditions, coacervation tends to occur, resulting in particles) and in the dispersion of nanoparticles in polymer matrices. 

Without a plasticizer, biopolymer materials are usually brittle due to a crosslinked network of densely hydrogen-bonded chains. Plasticizers help to break the original hydrogen bonds and the crystalline structure in biopolymers and to endow the produced biopolymer materials with flexibility and elasticity by acting as spacers between chains, limiting polymer chain interactions, and increasing chain mobility. Glycerol is the most widely used plasticizer for biopolymers due to its non-volatility and matching hydrophilicity [20,30,31]. Isosorbide, a bio-based plasticizer, has been studied to a much lesser extent, e.g., for poly(lactic acid) [32] and starch [33,34,35]. Compared with glycerol, isosorbide shows a great plasticizing effect for starch even at lower processing temperatures. Isosorbide has been found to retard starch retrogradation and make starch material properties more stable [33,34,35]. Other biopolymers such as chitosan and gelatin materials plasticized by isosorbide have scarcely been reported. Moreover, there has been limited understanding of how plasticizers such as glycerol and isosorbide affect chain interactions (hydrogen bonding and PEC) in dual-biopolymer systems, and the reinforcement effect of GO or MMT for plasticized biopolymer materials has not been thoroughly investigated. Previous findings have shown that polyols such as glycerol may negatively impact the dispersion of nanoclays or polysaccharide nanoparticles in plasticized biopolymer materials [20]. 

Our previous effort has led to the establishment of polyelectrolyte complexed chitosan–gelatin materials prepared by thermomechanical processing with excellent properties [19]. We found that a certain ratio of chitosan to gelatin (1:1) resulted in the lowest water absorption, best mechanical properties, and the highest *T*_g_ values. This enhanced performance could be attributed to the strong interactions (e.g., ionic and hydrogen bonding) between the polysaccharide and gelatin. Furthermore, adding gelatin assisted the processing of chitosan, while the composite films with a higher gelatin content were brighter, more transparent, and thus had a better visual appearance. Based on this, the goal of this work is to understand the effects of plasticizers (glycerol vs. isosorbide) and 2D nanofillers (MMT vs. GO) on the structure and properties of the 1:1 (*w*/*w*) chitosan–gelatin materials. Our hypothesis is that, in such a polyelectrolyte complexed system, the effects of plasticizer and nanofiller on material properties are mainly through influencing the PEC between the two reversely charged biopolymers. Understandings gained through testing this hypothesis could provide insights into the rational design of polymer composites involving both hydrogen-bonding and ionic interactions with desired material performance.

## 2. Materials and Methods

### 2.1. Materials

Chitosan, derived from crustaceous shells, with a specification of BR, was purchased from Xinhong Huagong Co., Ltd (Jinan, China) (moisture content of 13.01 wt%). This chitosan has a molecular mass of about 150,000 g∙mol^−1^, a degree of deacetylation of 90%, and a viscosity of about 400 mPa·s (1% solution in 1% acetic acid at 25 °C). The isoelectric point (pI) of chitosan is about 7.5 (see Figure S1). Food-grade gelatin (type A, bloom index 250, pI ≈ 4.8, see Figure S1) was supplied by Rousselot Gelatin Co., Ltd (Wenzhou, China) (moisture content of 10.75 wt%). Chemicals and materials used in this work also include isosorbide from Macklin (Shanghai, China), glycerol (AR) from Sinopharm Chemical Reagent Co., Ltd (Beijing, China), and acetic acid (AR) from Qiangsheng Functional Chemistry Co., Ltd (Jiangsu, China), MMT Nanomer^®^ PGV (aspect ratio: 150–200) from Nanocor Inc. (Chicago, IL, USA), and GO from Suiheng Tech (Shenzhen, China).2.2. Methods

### 2.2. Sample Preparation

**Figure 1:**
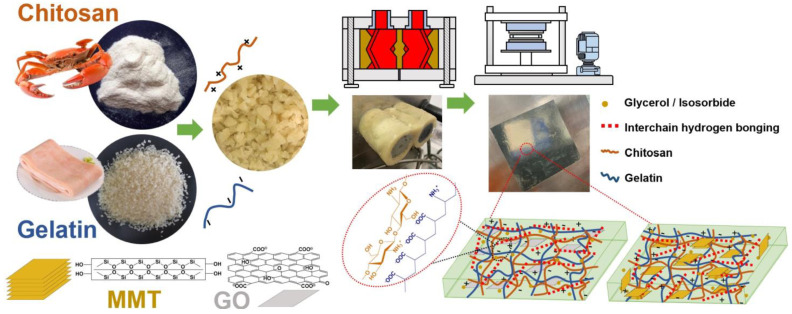
Detailed process and schematic illustration of the fabrication of chitosan–gelatin composite films.

Biopolymer films were prepared with their formulations and codes shown in Table 1. The sample codes such as “G-MMT-0.5” and “I-MMT-0.5” were used, where “G” and “I” represent glycerol and isosorbide as the plasticizer added to the biopolymer matrix, respectively, while “MMT-0.5” indicates the added mass content (0.5 wt%) of MMT (based on chitosan and gelatin). MMT or GO powder was dispersed in 2 M acetic acid solution (1% *w*/*v*, pH = 2.23) by ultrasonication for 30 min at 800 W and 19–23 kHz (JRA-1200X sonicator, Jieruian, China). Chitosan and gelatin were pre-blended mechanically for 15 min, during which the treated nanofiller suspensions and 2 M acetic acid solution were added dropwise. The total amount of 2 M acetic acid solution was 2.5 times that of chitosan and gelatin in dry mass minus the water and glycerol contents in the biopolymers. Then, the pre-blended chitosan–gelatin samples were stored overnight at 4 °C. For each batch of thermo-mixing, 80 g of one of the premixed samples was fed into a twin-rotor HAAKE Rheomix (Polylab RC600p system, ThermoHaake, Karlsruhe, Germany) and the processing was carried out for 15 min (G-GO samples for 12 min) at a screw speed of 80 rpm and a temperature of 80 °C. After that, 35 g of the thermally processed material was hot-pressed (80 °C, 2500 psi (17 MPa), 10 min) into films by a flat sulfuration machine (SY-6210-B, Shiyan, China) using a mold with a 100 mm × 100 mm × 1 mm hollow molding space. Then, the films were soaked in methanol for 12 h and then washed with deionized water (see Figure S2 for materials without and with methanol soaking). The detailed process of preparing chitosan–gelatin composite films is shown in Figure 1. All the specimens were dried in an oven at 30 °C for 24 h and then stored in a desiccator (57% relative humidity (RH)) for 4 weeks, followed by conditioning at either 57% RH or 75% RH for another week before characterization. 

### 2.3. Characterization Methods

#### 2.3.1. Scanning Electron Microscopy (SEM)

The sectional morphologies of the chitosan–gelatin composite films were imaged using an SEM facility (Phenom, Eindhoven, Netherlands) operated at a voltage of 10 kV. The cryo-fractured sectional surfaces were obtained by liquid nitrogen, and coated with gold for 90 s using a Q-150R-S sputter-coater (Quorum Technologies Ltd, East Sussex, UK).

#### 2.3.2. Fourier-Transform Infrared (FTIR) Spectroscopy

An FT-IR spectrometer Spectrum-3 (PerkinElmer, Mid Glamorgan, UK) fitted with a Zn-Se attenuated total reflectance (ATR) accessory was used to collect the infrared spectra for the samples. The spectral range was 4000–600 cm^−1^ and the spectral resolution was 4 cm^−1^.

#### 2.3.3. X-ray Diffraction (XRD) Analysis

An Xpert PRO diffractometer (Bruker, Karlsruhe, Germany) with a Cu Kα radiation (λ = 0.15418 nm) source operating at 40 mA and 40 kV was used. The scanning diffraction angle (2θ) spanned from 5° to 50° with a scanning speed of 2.16 s/step and a scanning step of 0.02°.

#### 2.3.4. Dynamic Mechanical Thermal Analysis (DMTA)

A DMA 8000 instrument (PerkinElmer, Mid Glamorgan, UK) was used to evaluate the dynamic mechanical thermal properties of the films (length of 20mm) with tensile mode. The tests were carried out from −50 °C to 150 °C at a heating rate of 2 °C/min, a fixed frequency of 1 Hz, and an amplitude of 10 μm. The dynamic storage modulus (E′), loss modulus (E″), and loss tangent (tan δ = E″/E′) were obtained. A thin layer of silicone oil was coated onto the samples to prevent water evaporation.

#### 2.3.5. Thermogravimetric Analysis (TGA)

An STA 8000 (PerkinElmer, Mid Glamorgan, UK) facility was used to evaluate the thermal stability of the samples in a nitrogen atmosphere over a temperature ramp from 30 °C to 650 °C at 10 °C/min. 

#### 2.3.6. Tensile Testing

The tensile properties of the films were evaluated according to ASTM D882-18. A tensile testing Instron 5566 apparatus (Instron, High Wycombe, UK) with a 100 N load cell was used for tensile testing at a crosshead speed of 5 mm/min. For each sample, the data were generated based on seven specimens.

#### 2.3.7. Water Absorption Capacity (WAC)

The water absorption was evaluated by the measurement of the weight change of the films (2 cm × 2 cm) after immersion in distilled water under ambient temperature (25 °C). Specifically, the films were then taken out at intervals, wiped with Whatman filter paper to remove the excess water, and weighed. The water absorption of the specimen was expressed as follows:

(1) $$\mathrm{WAC}\;\left(\%\right)=\frac{M_{t}-M_{0}}{M_{0}}\times 100$$

where *M*_0_ is the initial weight and *M_t_* is the weight after soaking in water for a certain time (*t*).

To evaluate the adsorption mechanism, the adsorption process was studied according to the two kinetics models [36]:

Pseudo-first order (PFO) model:

(2) $$q_{t}=q_{e\;}\left(1-e^{-K_{1}t}\right)$$

Pseudo-second order (PSO) model:

(3) $$\frac{t}{q_{t}}=\frac{1}{K_{2\;}q_{e}^{2}}+\frac{1}{q_{e}}t$$

where *K*_1_ (h^−1^) and *K_2_* (g·g^−1^·h^−1^) are the rate constants of the PFO and PSO models, respectively; *q_e_* and *q_t_* (g·g^−1^) are the amounts of the water adsorbed at an equilibrium state and at time *t*, respectively.

#### 2.3.8. Water Contact Angle (WCA)

The contact angle of water droplets on the films after 0 s and 60 s was measured using a contact angle system ZJ-7000 (Z. Jia Equipment, Shenzhen, China) at room temperature. Three drops of water (3 μL) were measured at different places on each sample. For each sample, four different places were measured.

## 3. Results and Discussion

### 3.1. Morphology

**Figure 2:**
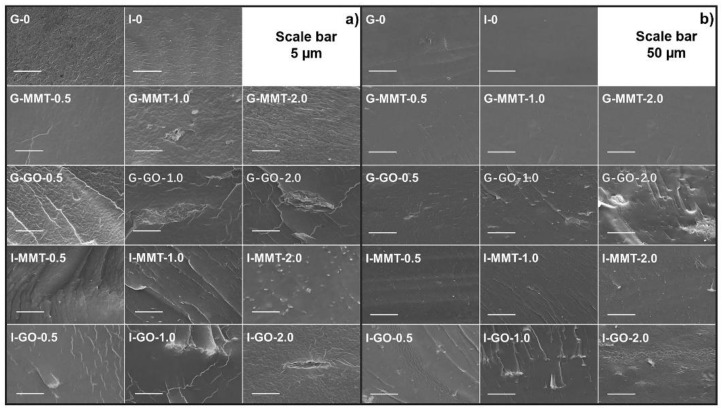
SEM images of cryo-fractured surfaces of the chitosan–gelatin films: (**a**) the scale bar represents 5 µm; and (**b**) the scale bar represents 50 µm. The samples were conditioned at 57% RH. All figures and tables should be cited in the main text.

Figure 2 shows the SEM images of cryo-fractured surfaces of the different biopolymer films. All the samples showed a relatively smooth, cohesive fractured surface, indicating the excellent plasticization and processing of the biopolymers. The surface morphologies do not display any discernible phase separation between chitosan and gelatin on the scales of observation, indicating a good compatibility and high interaction between the two biopolymers irrespective of the plasticizer type. 

I-MMT samples (especially I-MMT-2.0) presented more agglomerations of MMT nanoparticles than the G-MMT group (shown by white dots), indicating MMT nanosheets were dispersed better in glycerol-containing biopolymer matrices. However, there seemed to be more agglomerations of GO sheets in glycerol-plasticized samples than in isosorbide-plasticized ones. In this sense, the extent of dispersion of nanofillers in biopolymers depends on the plasticizer used. This difference could be linked to the viscosity of the plasticized biopolymers during processing (higher shear stress provides better a delamination effect on the nanosheets), and the interaction between the plasticizer and biopolymer chains, and the nanosheets. Isosorbide has a higher viscosity than glycerol at 80 °C (see Figure S3). When an overall material has a higher viscosity, there could be stronger shear facilitating the dispersion of nanoparticles in the matrix. However, the strong interaction between a plasticizer and nanoparticles may make nanoparticles agglomerate (as in the cases of G-GO and I-MMT films).

### 3.2. Molecular Interactions

**Figure 3:**
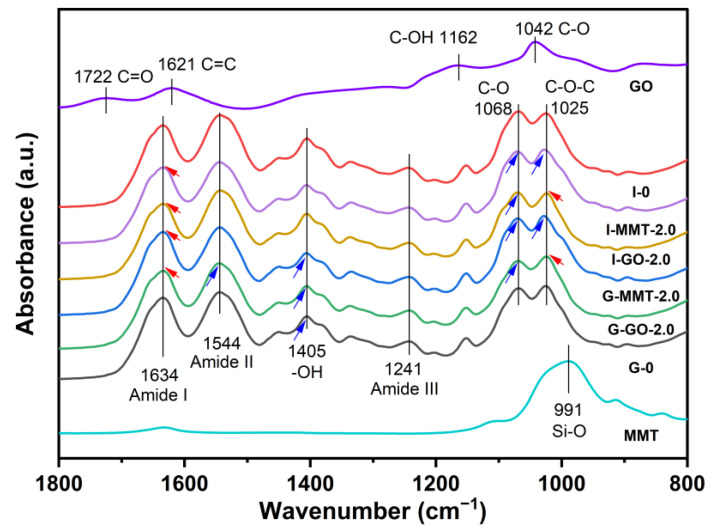
FTIR spectra for the different chitosan–gelatin films. The samples were conditioned at 57% RH. The reference lines for the films are based on the characteristic peaks of I-0.

Figure S4 shows FTIR spectra in the full range of testing for the different samples, and the enlarged spectra in the range of 1800–800 cm^−1^ were shown in Figure 3. G-GO, I-GO, G-MMT, and I-MMT films all displayed quite similar FTIR spectra as that of the chitosan–gelatin film without a nanofiller or plasticizer [19]. This suggests that addition of the nanofillers or the plasticizers did not cause any chemical derivation of the biopolymers. MMT displayed a sharp peak at 991 cm^−1^ due to the Si─O silica stretching vibration [37]. The spectrum of GO showed absorption peaks at 1042, 1162, 1621, and 1722 cm^−1^, which correspond to the stretching vibration of C─O, C─OH, C═C, and C═O, respectively [38]. However, these characteristic bands of MMT and GO were not readily visible on the spectra for the biopolymer films with them added, most likely due to the low addition levels. 

Compared with isosorbide-plasticized films, glycerol-plasticized ones showed the absorption peak of ─OH (at 1405 cm^−1^) appearing at a higher wavenumber position (blue shift), indicating there were stronger molecular interactions between glycerol and the biopolymers [19].

After the addition of GO, the two bands originally at 1544 cm^−1^ (N─H bending from amine and amide II) and 1068 cm^−1^ (asymmetric C─O─C stretching in the glycosidic linkage) were blue-shifted and there were two redshifts at 1025 cm^−1^ (C─O stretching) and 1634 cm^−1^ (amide I) [39,40]. For MMT-loaded biopolymers, two blue shifts (1068 cm^−1^ for C─O─C bonds and 1025 cm^−1^ for C─O bond) and one redshift (1634 cm^−1^ for amide I) were observed. The changes should be caused by the interactions (hydrogen bonding and ionic interaction) of GO or MMT with the biopolymer chains.

### 3.3. Crystalline Structure

**Figure 4:**
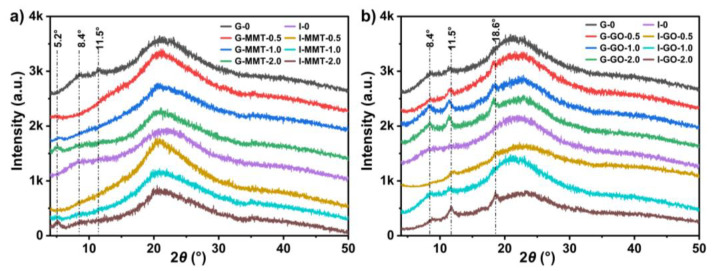
X-ray diffractograms for (**a**) the chitosan–gelatin films without and with MMT (The reference lines for the films are based on the characteristic peaks of G-0) and (**b**) the chitosan–gelatin films without and with GO (The reference lines for the films are based on the characteristic peaks of I-GO-2.0). The samples were conditioned at 57% RH.

The crystalline structures of the different biopolymer films were obtained by XRD, shown in Figure 4. Compared with the raw chitosan, which is semicrystalline (with diffraction peaks at around 2*θ* = 10.6° (0 2 0 reflection) and 20.1° (1 0 0 reflection)) (see Figure S5a), all the samples were mostly amorphous, with the disappearance of the two original characteristic peaks of the raw chitosan and the appearance of some weak peaks and an amorphous halo centered around 2*θ* = 22.5°. These results were similar to our previous reports that gelatin assisted the processing of chitosan and may also hinder the recrystallization of chitosan during conditioning [19]. While G-0 showed weak peaks at about 8.4° and 11.5°, these peaks were completely missing for I-0, suggesting isosorbide has a stronger capability of inhibiting chitosan recrystallization. 

The XRD patterns for the samples containing MMT showed the characteristic reflection peak of MMT at about 2*θ* = 5.2° (see Figure 4a), corresponding to an interlayer spacing (*d*_001_) of 1.70 nm. In comparison, the original MMT had a characteristic reflection peak at 2*θ* = 6.2°, corresponding to a *d*_001_ of 1.4 nm (see Figure S5b). The higher *d*_001_ value indicates that the composite biopolymer had good miscibility with MMT in this system and can easily intercalate into the interlayers through cationic exchange [41]. This corresponds well to the SEM observation of well-processed G-MMT samples. Compared with G-0, G-MMT samples showed no peaks at 2*θ =* 8.4° and 11.5°, suggesting that the presence of MMT nanosheets effectively suppressed chitosan recrystallisation. Nonetheless, inclusion of GO increased the reflection peaks at about 2*θ* = 8.4°, 11.5°, and 18.6°. A similar phenomenon was also found for chitosan/GO nanocomposites prepared by solution casting and was considered to be a result of GO-induced chitosan recrystallization [23,42]. The electrostatic interaction and hydrogen bonding between chitosan and GO may contribute to a relatively ordered arrangement of chitosan chains.

### 3.4. Relaxation Temperatures

**Figure 5:**
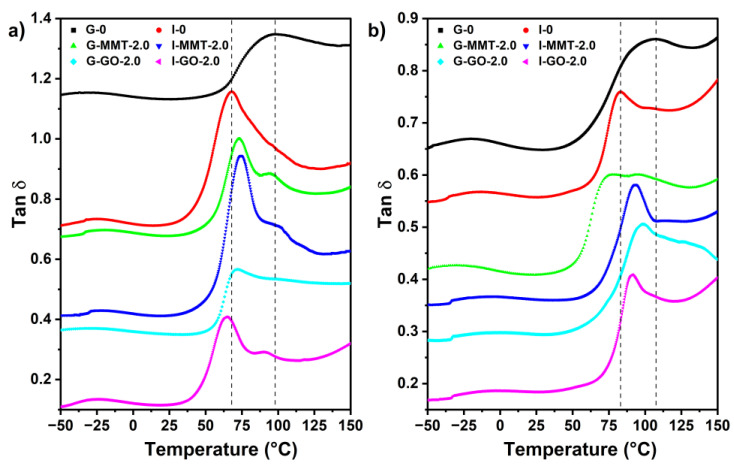
DMTA curves for the different chitosan–gelatin films at (**a**) 75% RH and (**b**) 57% RH. The reference lines are based on the *T*_g_ values of I-0 and G-0, respectively.

The molecular relaxation of the composite biopolymer films was evaluated using DMTA based on their loss tangent (tan *δ*) profiles as a function of temperature, shown in Figure 5. All the samples exhibit similar tan *δ* profiles with two transitions. The weak transition below 25 °C is associated with a β-relaxation attributed to the motions of the side chains or lateral groups of chitosan interacting with low-molecular-mass substances (water, glycerol, and isosorbide) typically by hydrogen bonding. After 25 °C, there was another much larger transition, representing an α-relaxation, which is considered the glass transition of the samples [12].

Among the samples conditioned at 75% RH, compared with G-0 (glass transition temperature *T*_g_ = 98.03 °C), both G-MMT-2.0 and G-GO-2.0 displayed lower *T*_g_ values (73.16 °C and 72.20 °C, respectively). Given this, it is likely that the MMT/GO nanosheets assisted the distribution of glycerol and water, and/or the retention of water, in the biopolymer matrix and thus enhanced chain mobility. Compared with G-0, I-0 had a lower *T*_g_ value (68.19 °C), indicating isosorbide had a stronger plasticization effect (i.e., reducing biopolymer chain interactions). As the biopolymer matrix is composed of chitosan and gelatin, which could form PEC, it is proposed that isosorbide may also inhibit PEC between chitosan and gelatin more effectively, which could also increase chain mobility. Inclusion of MMT into the isosorbide-plasticized matrix at 2.0 wt% loading decreased the chain mobility (*T*_g_ = 74.45 °C) but I-GO-2.0 displayed increased chain mobility (*T*_g_ = 64.51 °C). The dispersion of nanosheets in the biopolymers may restrict chain mobility (increasing *T*_g_) and facilitate the distribution of the plasticizer and water in the matrix and thus enhance chain mobility (decreasing *T*_g_). Presumably, MMT and GO have different extents of these two effects on the isosorbide-plasticized matrix conditioned at 75% RH.

Compared with the samples conditioned 75% RH, the 57% RH samples generally showed higher *T*_g_ values. This is as expected as the lower moisture contents in the 57% RH samples mean reduced chain mobility. The *T*_g_ values of G-0 and I-0 are 106.25 °C and 82.95 °C, respectively, and the better plasticization effect of isosorbide was evident again. Compared with G-0 conditioned at 57% RH, G-MMT-2.0 and G-GO-2.0, again, showed lower *T*_g_ values (95.65 °C and 98.52 °C, respectively), likely due to more homogenously distributed glycerol and water, or the greater retention of water, in the matrix and thus improved plasticization. Compared with I-0 conditioned at 57%, both I-MMT-2.0 and I-GO-2.0 showed higher *T*_g_ values (93.31 °C and 91.22 °C, respectively), presumably caused by the restricted chain mobility with the dispersion of the nanosheets. Irrespective of RH, G-GO-2.0 exhibited higher *T*_g_ values than I-GO-2.0, which can be attributed to the higher crystallinity of G-GO-2.0 film restricting the segmental mobility of biopolymer chains (see XRD results) [43].

Regardless of formulation and RH, the *T*_g_ values of all the samples were well above room temperature, indicating that they were all in a glassy state.

### 3.5. Mechanical Properties

**Figure 6:**
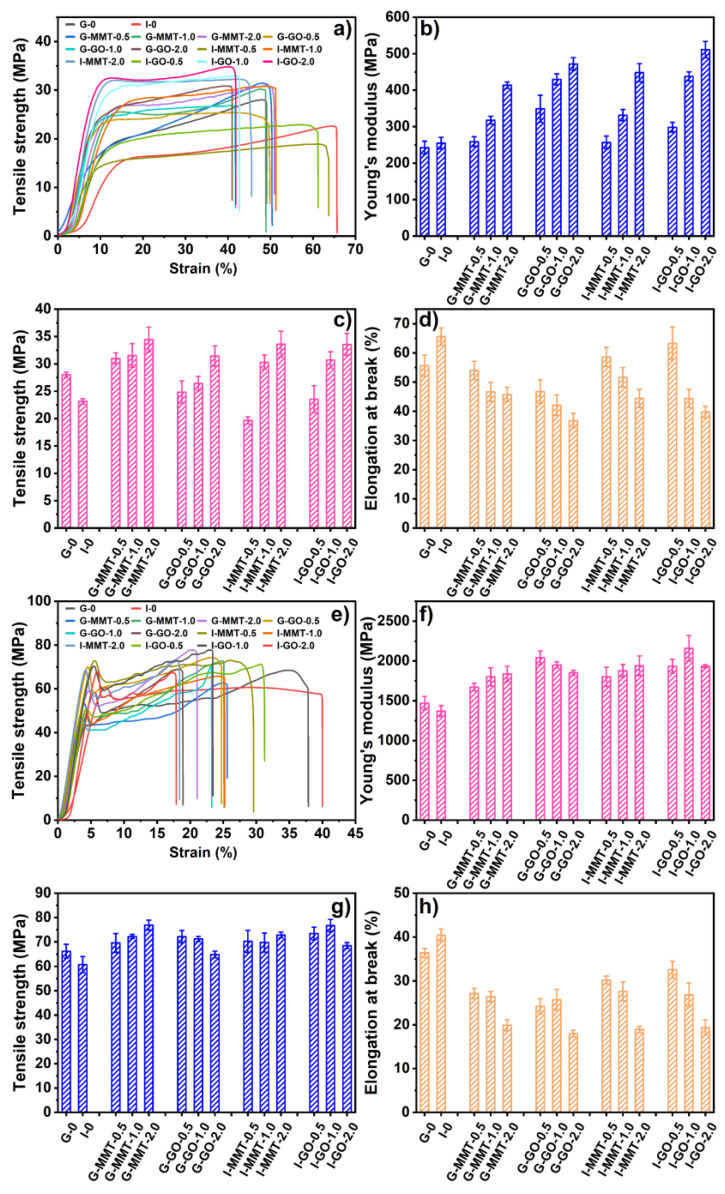
Mechanical properties of the chitosan–gelatin films at 75% RH (**a**–**d**) and 57% RH (**e**–**h**). The error bars represent standard deviations.

Figure 6 shows the Young’s modulus (*E*), tensile strength (*σ*_t_), and elongation at break (*ε*_b_) of the different biopolymer films at 57% RH and 75% RH. All the films after conditioning showed a typical stress–strain curve of a hard and tough polymer, with strain-hardening behavior, indicating strong interactions between biopolymer chains restricting slide from each other. The stress–strain curve also shows the rigidity of the materials, as all of them were in a glassy state (as shown by the DMTA results). Inclusion of MMT or GO influenced the *E*, *σ*_t_, and *ε*_b_ parameters of the biopolymer films irrespective of plasticizer and RH. 

Among the samples conditioned at 75% RH, I-0 (*E* = 254.9 ± 16.1 MPa, *σ*_t_ = 23.2 ± 0.5 MPa, *ε*_b_ = 65.60 ± 3.0%) was more flexible than G-0 (*E* = 242.6 ± 17.7 MPa, *σ*_t_ = 28.0 ± 0.49 MPa, *ε*_b_ = 55.61 ± 3.6%). This could indicate that isosorbide has a stronger plasticization effect (i.e., reducing biopolymer chain interactions and the PEC between chitosan and gelatin) than glycerol for biopolymers, as discussed before for the DMTA results. Generally, the plasticized samples with a higher content of MMT or GO displayed higher *E* and *σ*_t_ and lower *ε*_b_, showing the reinforcement effect of these nanofillers. In particular, G-MMT-2.0 (*E* = 435.7 ± 9.0 MPa, *σ*_t_ = 34.4 ± 2.3 MPa, *ε*_b_ = 45.8 ± 2.4%), G-GO-2.0 (*E* = 471.9 ± 17.4 MPa, *σ*_t_ = 32.4 ± 1.9 MPa, *ε*_b_ = 36.83 ± 2.5%), I-MMT-2.0 (*E* = 484.4±13.4 MPa, *σ*_t_ = 33.6±2.4 MPa, *ε*_b_ = 44.4±3.1%), and I-GO-2.0 (*E* = 511.4 ± 22.3 MPa, *σ*_t_ = 33.6 ± 2.3 MPa, *ε*_b_ = 39.76 ± 2.0%) displayed the best mechanical properties among the 75% RH samples. Regarding this, the interactions between MMT/GO and chitosan/gelatin chains can lead to a mechanism for effectively transferring the interfacial stress, and the well-dispersed MMT/GO nanosheets in the biopolymer matrix can facilitate uniform stress distribution and minimize the presence of stress concentration, which contributed to the improved mechanical properties of the biopolymers [11,12]. However, inclusion of MMT or GO at 0.5 wt% loading did not provide a strong reinforcement effect or even reduce mechanical properties (the *σ*_t_ values of G-MMT-0.5, G-GO-0.5, I-MMT-0.5, and I-GO-0.5 were 31.0 ± 1.0 MPa, 24.8 ± 2.1 MPa, 19.7 ± 0.7 MPa and 23.6 ± 2.4MPa, respectively). Regarding this, it is likely that when the MMT/GO content was low, their effect was mainly through facilitating the dispersion of the plasticizer in the biopolymer matrix, enhancing the plasticization effect.

Compared with the samples conditioned at 75% RH, those conditioned at 57% RH were more rigid, with much higher *E* and *σ*_t_ and lower *ε*_b_. Unlike the 75% RH samples, the 57% RH samples displayed an obvious yield point on the stress–strain curve. These suggest that the moisture content in the samples significantly affected the mechanical properties, which is sensible. A reduced content of moisture in biopolymer materials could reduce the space between biopolymer chains and lead to strengthened chain interactions. Compared with G-0 (*E* = 1470.3 ± 84.0 MPa, *σ*_t_ = 66.2 ± 2.9 MPa, *ε*_b_ = 36.4 ± 1.0%), I-0 displayed slight decreases in these mechanical properties (*E* = 1370.1 ± 69.2 MPa, *σ*_t_ = 60.8 ± 3.3 MPa, *ε*_b_ = 40.4 ± 1.4%), indicating, again, isosorbide has a strong plasticization effect (i.e., reducing biopolymer chain interactions). Compared with G-0 and I-0, the plasticized samples included with MMT or GO all showed increased mechanical properties and higher rigidity (higher *E* and *σ*_t_ and lower *ε*_b_). Generally, an increasing content (from 0.5 to 2.0 wt%) of MMT further increased the *E* and *σ*_t_ and reduced *ε*_b_ of the chitosan–gelatin material irrespective of the plasticizer type. However, when GO was used as the nanofiller at the highest content (2.0 wt%), the mechanical properties were even lower than those with lower contents of GO. Regarding this, GO agglomeration probably caused weak points in the material. G-MMT-2.0 and I-GO-1.0 displayed the highest *σ*_t_ values (76.8 ± 2.0 MPa and 76.6 ± 2.5 MPa, respectively). This was so despite the fact that a higher content of GO slightly increased the crystallinity of chitosan (see the XRD results), which also suggests that the crystallinity of the materials was too low to have a major effect on mechanical properties.

### 3.6. Thermal Stability

**Figure 7:**
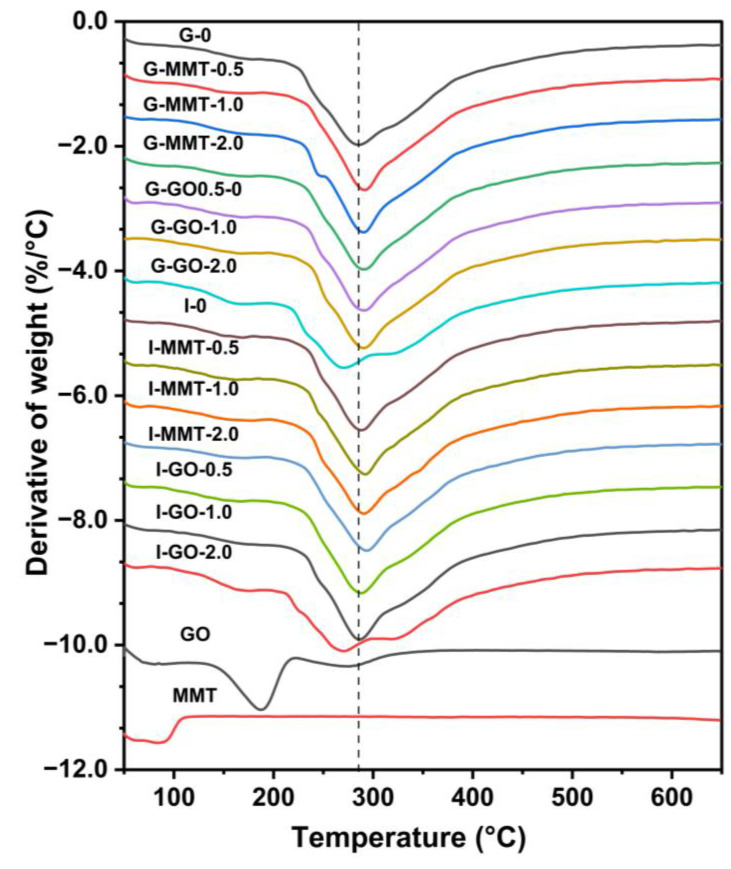
Derivative TGA results for the different chitosan–gelatin films, raw GO, and raw MMT. The samples were conditioned at 57% RH. The reference line is based on the *T*_d_ of G-0.

Figure 7 shows the derivative TGA results for the raw GO, the raw MMT, and the different chitosan–gelatin composite films. For the raw GO, there was a major weight loss between 115 °C and 215 °C with the peak temperature (*T*_d_, at the maximum weight-loss rate) at about 181 °C, accompanied by a small, broad peak between 215 °C and 370 °C, presumably due to pyrolysis of the unstable oxygen-containing functional groups [44]. MMT only showed an initial weight loss due to water evaporation and remained thermally stable until the highest test temperature (650 °C).

Compared with G-0 and I-0 (*T*_d_ = 286 °C and 288 °C, respectively), the samples containing MMT were more thermally stable (*T*_d_ = 291 °C for G-MMT-2.0, and 294 °C for I-MMT-2.0), indicating that inclusion of MMT was effective at enhancing the biopolymer thermal stability in this system, irrespective of plasticizer type. Regarding this enhancement, the dispersed MMT nanosheets in the polymer matrix could act as a heat barrier and effectively retard the transfer to pyrolysis products (gasses and radicals).

When low GO loading levels were used, the samples added with GO (*T*_d_ = 291 °C, 290 °C, 290 °C, and 288 °C for G-GO-0.5, G-GO-1.0, I-GO-0.5, and I-GO-1.0, respectively) showed higher thermal stability compared to G-0 and I-0. Given this, it is likely that the well-exfoliated nanosheets can also impede the diffusion of thermal degradation products and increase the activation energy for thermal degradation. However, the samples included with GO at 2.0 wt% loading (*T*_d_ = 270 °C for G-GO-2.0 and *T*_d_ = 271 °C for I-GO-2.0) showed reduced thermal stability. It is proposed that when the GO content is high, the oxygen-containing functional groups of GO promote the thermal decomposition of chitosan and gelatin [24].

### 3.7. Water Absorption Capacity (WAC)

**Figure 8:**
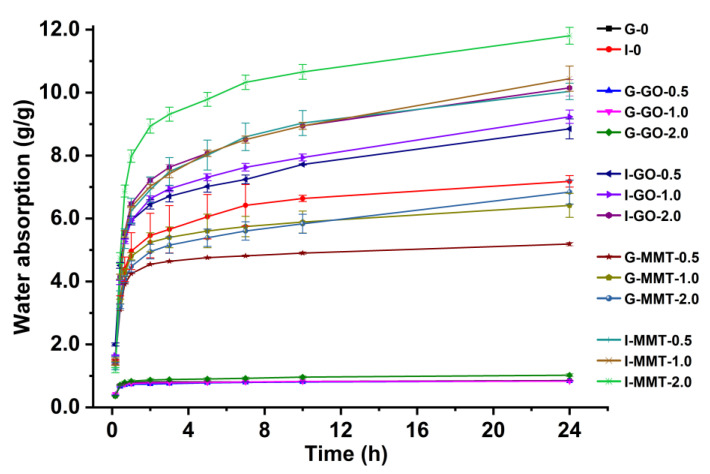
Water absorption as a function of time of the different biopolymer films. The error bars represent standard deviations.

Figure 8 shows that all the samples experienced a drastic increase in weight at the beginning of the water-soaking process (the first 2 h), followed by a much slower absorption rate. The samples using different plasticizers displayed a significant difference in the maximum WAC (within 24 h of testing), G-0 0.85 ± 0.08 g/g and I-0 7.18 ± 0.18 g/g. We can see I-0 was significantly swollen after water soaking, whereas there was a minor change to the original size (2 cm × 2 cm) of G-0 after water soaking as shown in Figure S6. This was so despite the fact that glycerol with more hydroxyl groups per molecule and a lower molecular mass should be more hydrophilic than isosorbide. We consider that the differences in water uptake and dimensional change are mainly associated with the PEC between chitosan and gelatin [12,19]. As electronic attraction is a type of force much stronger than hydrogen bonding, PEC stabilizes and inhibits the expansion of the biopolymer chain network although hydrogen bonds are disrupted by water. Our previous study indicated that compared with the chitosan–gelatin composite film without plasticizer, the glycerol-plasticized one only displayed a marginal increase in water uptake, which means glycerol does not have a detrimental effect on PEC [19]. In contrast, it is proposed that PEC between chitosan and gelatin could not be maintained with the presence of isosorbide, which explains the poorer hydrolytic stability of I-0 than G-0. 

The data show that the effect of nanofillers on the water uptake of the biopolymer materials depends on the plasticizer used. The maximum WAC of the films (at the same nanofiller addition levels) followed the sequence of I-MMT > I-GO > G-MMT > G-GO. The WAC of the I-MMT films increased from 10.04 ± 0.26 g/g to 11.81 ± 0.27 g/g with the MMT content increasing from 0.5 wt% to 2.0 wt%. The I-GO films absorbed less water than the I-MMT films at the same nanofiller levels. Interestingly, while the G-GO samples showed similar water uptake and dimensional change to G-0, the G-MMT samples appeared to be significantly more hygroscopic. This phenomenon suggests that when glycerol was used as a plasticizer, the addition of GO did not significantly vary the PEC between chitosan and gelatin, but the inclusion of MMT disrupted PEC and made the biopolymer chain network less hydrolytically stable. Nonetheless, the I-GO samples had greater WAC than I-0, suggesting that when isosorbide was present, GO further disrupted PEC and made the materials more hygroscopic. 

The water absorption of kinetics was studied following the PFO and PSO models to determine the rate-controlling process and the mechanism of adsorption, which are crucial properties for the design and practical use of chitosan–gelatin composite films. According to the parameters, as shown in Table 2, higher *R*^2^ values were obtained from the PSO model compared to the PFO model and closer to unity. Additionally, for all the samples, the calculated equilibrium adsorption capacities (*q*_e_) obtained from the PSO model were well approximated to the experimental WAC values. These results indicate that the PSO model more suitably described water adsorption kinetics for the chitosan–gelatin composite films, which should be related to not only the abundance of hydrophilic groups, but also electrostatic attraction among biopolymer chains. It was noted that isosorbide and MMT could significantly disrupt PEC between chitosan and gelatin polymer chains, which corresponds well with the greater hygroscopicity of the samples containing isosorbide and MMT.

### 3.8. Surface Hydrophilicity

**Figure 9:**
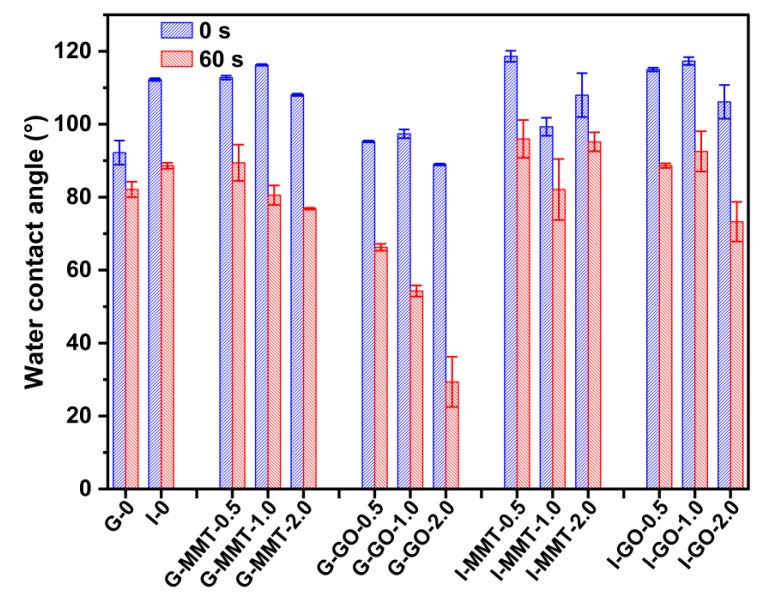
Water contact angle measured at 0 s and 60 s for the different films. The samples were conditioned at 57% RH. The error bars represent standard deviations.

A WCA test was conducted to investigate the surface hydrophilicity of the biopolymer films as shown in Figure 9. Table S1 shows the droplet images for the different samples at 0 s and 60 s. A lower WCA means higher surface hydrophilicity (higher surface free energy), which is primarily associated with the chemical groups exposed on the material surface [39]. All WCA values declined after a water drop was placed on the film surface for 60 s. Regarding the decreasing WCA with time, during wetting, water could disrupt biopolymer chain interactions on the material surface, leading to more free polar groups available to bind water [40]. In addition, the slight evaporation of water and the slow water uptake of the composite films (since both chitosan and gelatin are hydrophilic) may also lead to a slight decrease in WCA. It can be seen that I-0 (112.3° at 0 s and 88.6° at 60 s) had higher WCA than G-0 (92.2° at 0 s and 82.1° at 60 s), meaning the greater surface hydrophobicity of the former (although I-0 had greater water uptake as discussed above). We consider that the surface hydrophilicity is controlled by the number of high-energy groups exposed on the material surface while the water uptake is determined by the overall hygroscopicity and chain network structure of the material. For biopolymers such as gelatin, high chain mobility would allow for the burying of polar groups in the bulk phase, making the surface more hydrophobic [45]. Probably, isosorbide more effectively assisted chain mobility and facilitated the self-configuration of biopolymer chains on the material surface, leading to fewer polar groups exposed on the material surface. 

Compared with G-0, the G-MMT samples showed moderately increased WCA (especially at 0 s, 108.1–116.3°), while the G-GO samples displayed significantly decreased WCA (especially at 60 s, 29–66°). This trend is opposite to that of water uptake, which is interesting. This difference in WCA may be linked to the difference in hydrophilicity between MMT and GO. Additionally, it is possible that GO was effective at restricting chain rearrangement on the material surface, whereas MMT nanosheets provided a shielding effect for polar groups. However, this negative effect of GO on surface hydrophobicity for the isosorbide-plasticized biopolymers was not evident. Regarding this latter case, it is speculated that the effect of isosorbide on the chain rearrangement on the material surface should dominate over the influence of the nanofiller. While the effects of plasticizer and nanofiller on such dual-biopolymer systems are worth further investigation, it is confirmed here that the surface hydrophilicity/hydrophobicity is controlled by different mechanisms from those for the overall material water absorption. 

## 4. Conclusions

This work shows the different effects of plasticizers (glycerol and isosorbide) and 2D nanofillers (MMT and GO) on the properties of chitosan–gelatin composite materials. Both glycerol and isosorbide are effective plasticizers for biopolymers to suppress recrystallization, but isosorbide might additionally disrupt the PEC between chitosan and gelatin. For the changes in *T*_g_, mechanical properties, and water absorption, how the PEC between chitosan and gelatin was affected by plasticizer and nanofiller played a significant role. While, compared with isosorbide, glycerol should have stronger interaction with the biopolymers (indicated by FTIR), it is evident from the DMTA and tensile testing results that isosorbide has a stronger plasticization effect by disrupting biopolymer chain interactions (both hydrogen bonding and PEC). The effect of nanofiller addition on *T*_g_ and mechanical properties also probably depends on how the nanofiller influences plasticization. Additionally, while the glycerol-plasticized samples without nanofiller or with GO showed minimal water absorption and dimensional change in water, we found the addition of isosorbide and/or MMT could disrupt PEC and make the materials significantly swollen in water. However, WCA results show a reverse trend; namely, isosorbide-plasticized samples had greater surface hydrophobicity and addition of GO to the glycerol-plasticized biopolymer matrix increased surface hydrophilicity significantly, which should be more associated with surface chemistry. Moreover, this work also shows that inclusion of MMT or GO (except for GO at 2.0 wt% loading) as 2D nanofillers could generally enhance mechanical properties and thermal stability, which should be ascribed to their large surface area and excellent interfacial interaction with biopolymers. Thus, this work highlights different mechanisms (especially PEC, which has been less considered before) that control the different properties of dual-biopolymer nanocomposite systems, providing insights into the design of polymeric materials involving multiple ways of chain interaction with tailored properties.

## Figures and Tables

**Table 1:** Sample codes and composition.

Sample	Chitosan/Gelatin (g, Dry Weight)	Glycerol (g)	Isosorbide (g)	MMT Solution ^a^ (mL)	GO Solution ^a^ (mL)	2 M Acetic Acid (mL)
G-0	13.0/13.0 (100%)	3.9 (15%)	-	-	-	57.59
G-MMT-0.5	13.0/13.0	3.9	-	13 (0.5%)	-	44.59
G-MMT-1.0	13.0/13.0	3.9	-	26 (1.0%)	-	31.59
G-MMT-2.0	13.0/13.0	3.9	-	52 (2.0%)	-	5.59
G-GO-0.5	13.0/13.0	3.9	-	-	13 (0.5%)	44.59
G-GO-1.0	13.0/13.0	3.9	-	-	26 (1.0%)	31.59
G-GO-2.0	13.0/13.0	3.9	-	-	52 (2.0%)	5.59
I-0	13.0/13.0	-	3.9 (15%)	-	-	57.59
I-MMT-0.5	13.0/13.0	-	3.9	13 (0.5%)	-	44.59
I-MMT-1.0	13.0/13.0	-	3.9	26 (1.0%)	-	31.59
I-MMT-2.0	13.0/13.0	-	3.9	52 (2.0%)	-	5.59
I-GO-0.5	13.0/13.0	-	3.9	-	13 (0.5%)	44.59
I-GO-1.0	13.0/13.0	-	3.9	-	26 (1.0%)	31.59
I-GO-2.0	13.0/13.0	-	3.9	-	52 (2.0%)	5.59

^a^ MMT/GO dispersed in 2 M acetic acid solution (1% *w*/*v*). The percentages are based on the total dry weight of chitosan and gelatin matrix.

**Table 2:** Calculated parameters of kinetic studies and actual maximum water absorption of the films.

Sample	WAC (g/g)	Pseudo-First-Order			Pseudo-Second-Order		
		*K*_1_ (h^−1^)	*q*_e_ (g·g^−1^)	*R* ^2^	*K*_2_ (g·g^−1^·h^−1^)	*q*_e_ (g·g^−1^)	*R* ^2^
G-0	0.85 ± 0.08	3.64 ± 0.21	0.82 ± 0.01	0.9244	7.04 ± 1.34	0.85 ± 0.02	0.9847
I-0	7.18 ± 0.18	1.52 ± 0.06	6.73 ± 0.13	0.9924	0.23 ± 0.02	7.16 ± 0.18	0.9951
G-GO-0.5	0.84 ± 0.01	4.12 ± 0.38	0.78 ± 0.01	0.9391	8.68 ± 1.32	0.82 ± 0.02	0.9431
G-GO-1.0	0.83 ± 0.02	4.50 ± 0.33	0.80 ± 0.0	0.9599	9.02 ± 1.12	0.83 ± 0.01	0.9671
G-GO-2.0	1.02 ± 0.05	5.35 ± 0.19	0.91 ± 0.02	0.9480	4.23 ± 0.74	1.00 ± 0.04	0.9797
I-GO-0.5	8.85 ± 0.32	1.85 ± 0.15	7.68 ± 0.08	0.9881	0.29 ± 0.03	8.04 ± 0.08	0.9911
I-GO-1.0	9.23 ± 0.22	1.57 ± 0.11	7.48 ± 0.26	0.9779	0.18 ± 0.03	8.76 ± 0.45	0.9851
I-GO-2.0	10.15 ± 0.26	1.14 ± 0.10	8.47 ± 0.42	0.9630	0.10 ± 0.02	10.43 ± 0.80	0.9790
G-MMT-0.5	5.19 ± 0.06	2.74 ± 0.12	4.66 ± 0.06	0.9270	0.92 ± 0.15	5.11 ± 0.16	0.9735
G-MMT-1.0	6.42 ± 0.38	1.90 ± 0.15	5.79 ± 0.23	0.9556	0.30 ± 0.07	6.84 ± 0.47	0.9828
G-MMT-2.0	6.84 ± 0.38	2.06 ± 0.16	5.46 ± 0.19	0.9710	0.40 ± 0.06	6.20 ± 0.25	0.9718
I-MMT-0.5	10.04 ± 0.26	0.92 ± 0.10	9.19 ± 0.53	0.9613	0.08 ± 0.02	10.49 ± 0.70	0.9715
I-MMT-1.0	10.44 ± 0.40	1.61 ± 0.16	7.93 ± 0.28	0.9270	0.25 ± 0.04	9.04 ± 0.33	0.9519
I-MMT-2.0	11.81 ± 0.27	1.23 ± 0.18	10.40 ± 0.51	0.9553	0.11 ± 0.03	11.92 ± 0.80	0.9640

## Data Availability

The data presented in this study are available on request from the corresponding author.

## Supplementary Materials

The following supporting information can be downloaded at: https://www.mdpi.com/article/10.3390/polym14183797/s1, Figure S1: ζ-Potential *vs.* pH curves for chitosan and gelatin; Figure S2: (**a**) G-0 film after being soaked in methanol for 12 h and then in 0.1 M NaOH for another 12 h; (**b**) G-0 film after being soaked only in 0.1 M NaOH solution for 12 h; Figure S3: Viscosities of isosorbide and glycerol at 80 °C; Figure S4: FTIR spectra for the different chitosan–gelatin films; Figure S5: X-ray diffractogram of (**a**) raw chitosan, and raw gelatin; (**b**) montmorillonite (MMT) (the peak position corresponds to *d*_001_ = 1.4 nm); Figure S6: (**a**) Photos of different biopolymer films; (**b**) and the films after soaking in water for 24 h; Table S1: Contact angle images of the different chitosan–gelatin films.
