# Supplementary material for: Chitosan–Gelatin Films: Plasticizers/Nanofillers Affect Chain Interactions and Material Properties in Different Ways
Source: Polymers (Basel). 2022 Sep 11;14(18):3797. doi: 10.3390/polym14183797 (PMC9505206; doi:10.3390/polym14183797)
Supplement: Supplementary file 1 [file polymers-14-03797-s001.zip › polymers-1890991-supplementary.pdf]

Supplementary Material

Chitosan–Gelatin Films: Plasticizers/Nanofillers Affect Chain Interactions and Material Properties in Different Ways

Qingfei Duan [1,†], Ying Chen [2,*,†], Long Yu [1] and Fengwei Xie [3,*]

[1] College of Food Science and Engineering, South China University of Technology, Guangzhou 510640, China
[2] School of Food Science and Engineering, Yangzhou University, Yangzhou 225127, China
[3] School of Engineering, Newcastle University, Newcastle upon Tyne, NE1 7RU, United Kingdom
* Correspondence: 008265@yzu.edu.cn (Y.C.); david.xie@newcastle.ac.uk or fwhsieh@gmail.com (F.X.)
† These authors contributed equally to this work.

Number of pages: 5

Number of figures: 6

Number of tables: 1

1 Figures

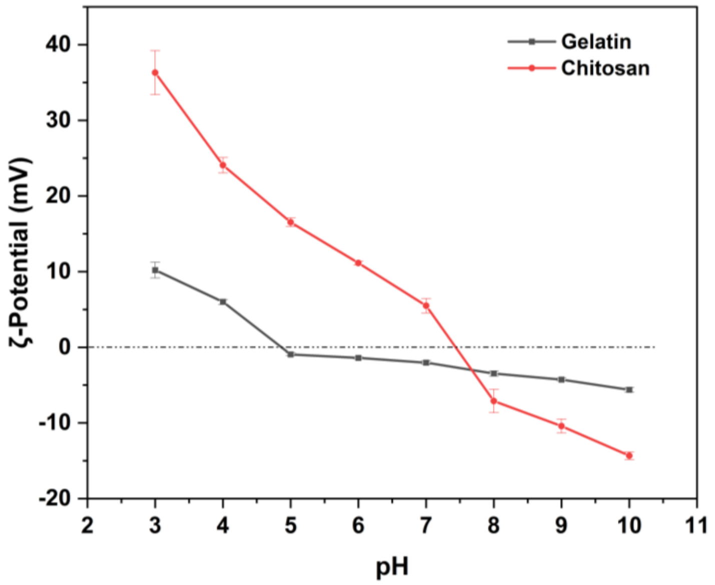

Figure S1. ζ-Potential *vs.* pH curves for chitosan and gelatin.

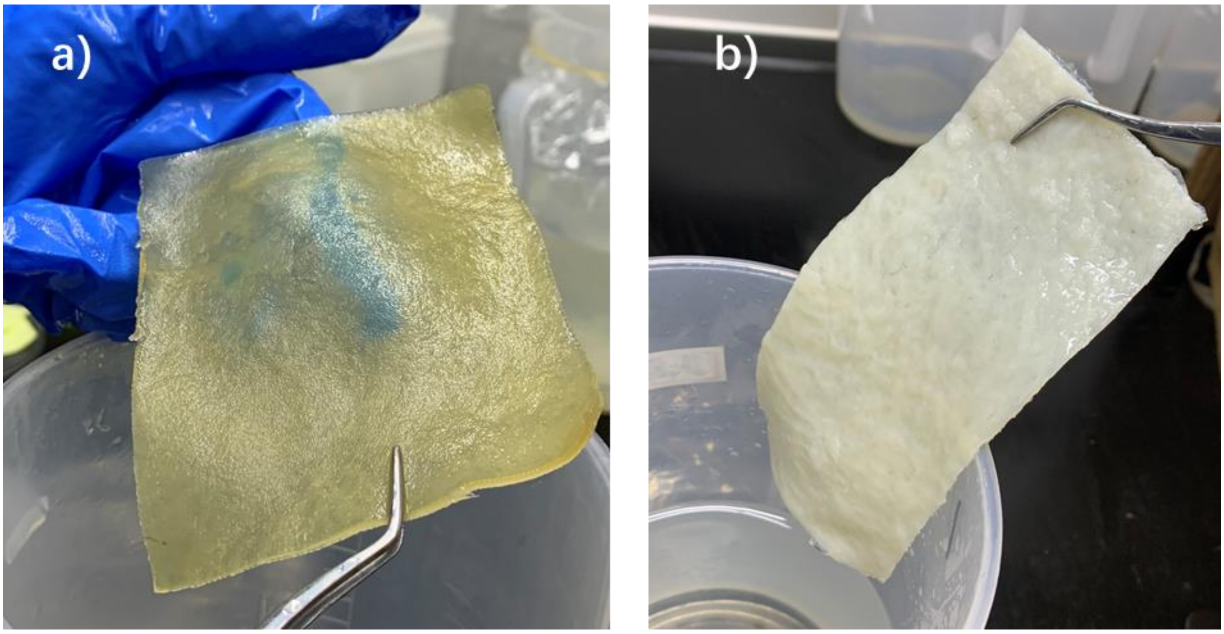

Figure S2. a) G-0 film after being soaked in methanol for 12 h and then in 0.1M NaOH for another 12 h; b) G-0 film after being soaked only in 0.1M NaOH solution for 12 h.

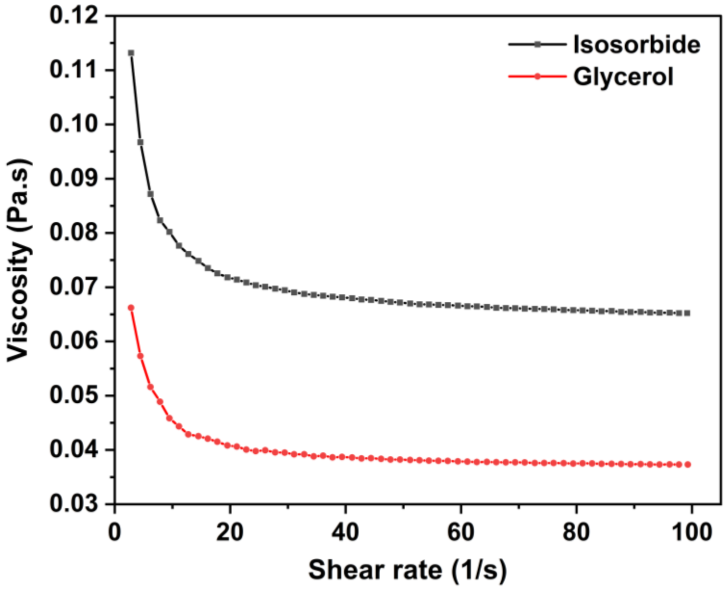

Figure S3. Viscosities of isosorbide and glycerol at 80 °C.

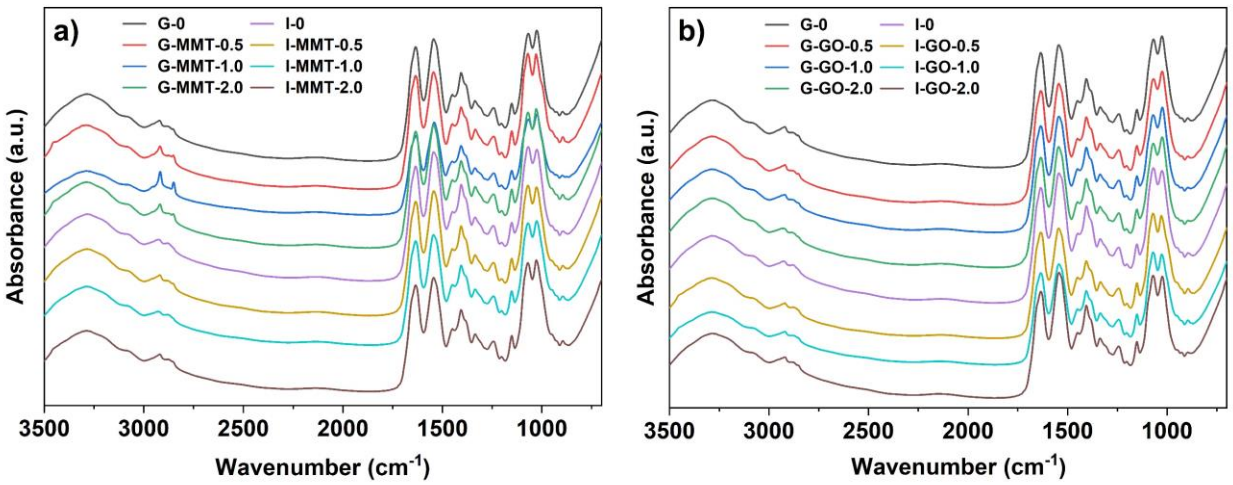

Figure S4. FTIR spectra for the different chitosan-gelatin films.

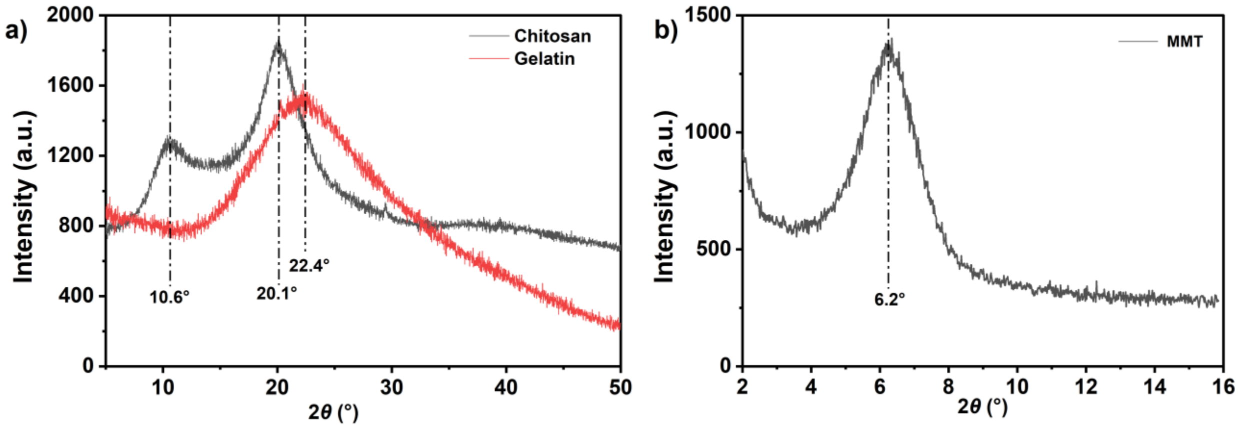

Figure S5. X-ray diffractogram of **a)** raw chitosan, and raw gelatin; **b)** montmorillonite (MMT) (the peak position corresponds to d_{001} = 1.4 nm).

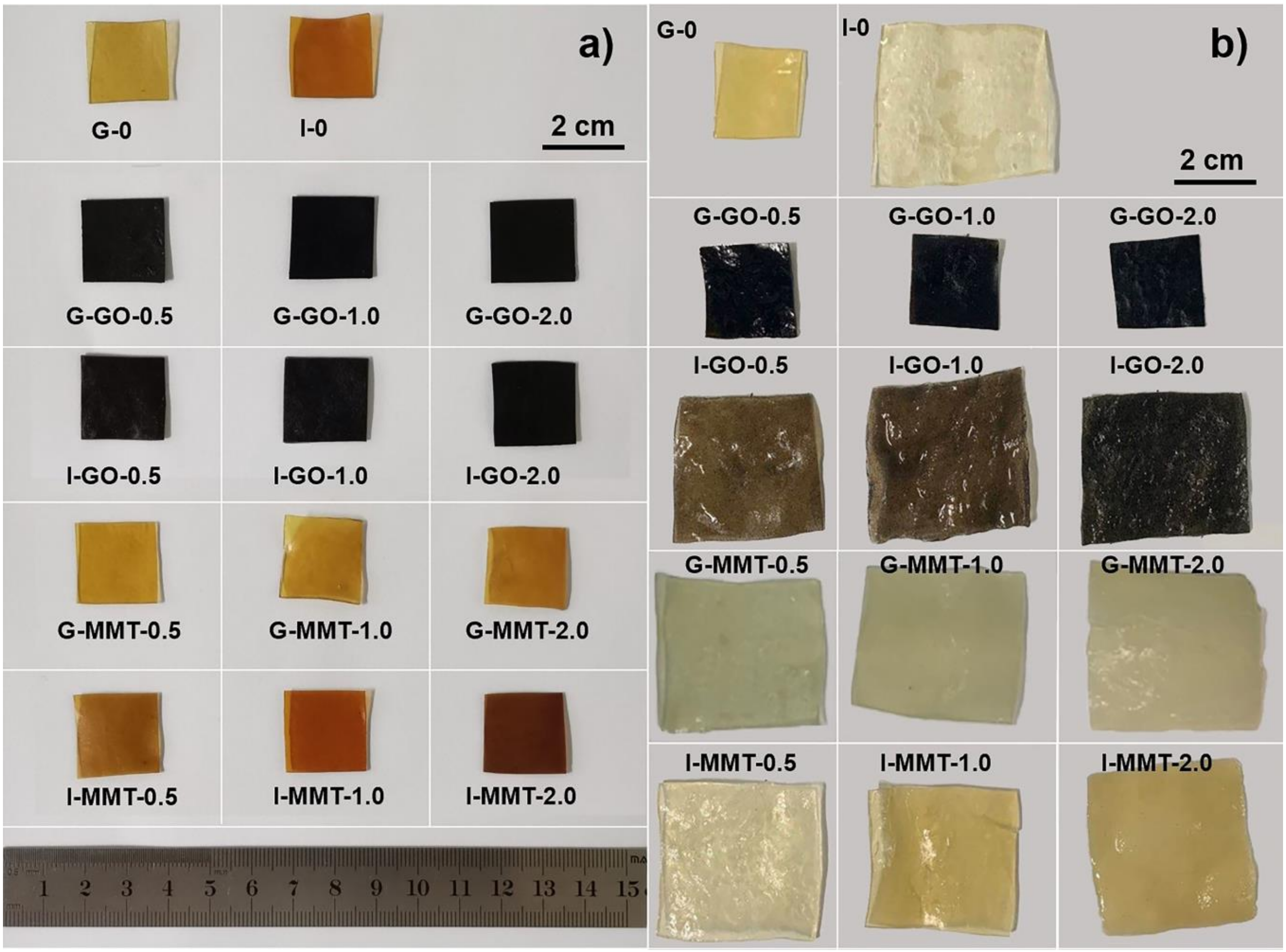

Figure S6. a) Photos of different biopolymer films; b) and the films after soaking in water for 24 h.

Table S1. Contact angle images of the different chitosan-gelatin films.

Films	WCA (°, t_0)	Image	WCA (°, t_{60})	Image
G-0	92.2±3.3	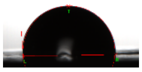	82.1±2.3	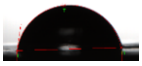
I-0	112.3±0.4	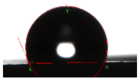	88.6±0.8	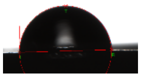
G-MMT-0.5	112.8±0.5	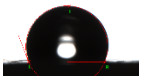	89.4±4.9	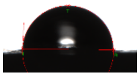
G-MMT-1.0	116.3±0.2	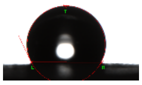	80.6±2.6	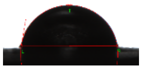
G-MMT-2.0	108.1±0.3	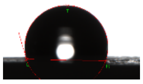	76.8±0.2	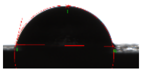
G-GO-0.5	95.3±0.3	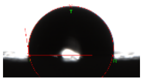	66.3±0.9	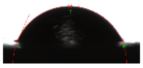
G-GO-1.0	97.4±1.2	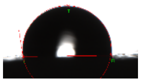	54.3±1.5	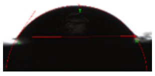
G-GO-2.0	89.0±0.3	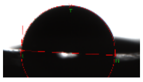	29.4±6.8	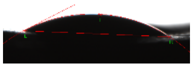
I-MMT-0.5	118.6±1.5	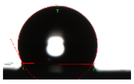	96.0±5.2	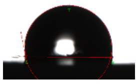
I-MMT-1.0	99.3±2.5	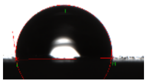	82.1±8.4	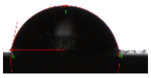
I-MMT-2.0	108.0±6.0	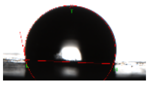	95.2±2.6	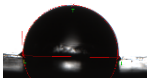
I-GO-0.5	115.0±0.5	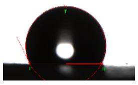	88.6±0.6	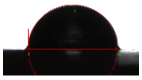
I-GO-1.0	117.3±1.1	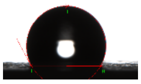	92.54±5.5	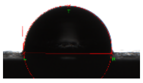
I-GO-2.0	106.1±4.6	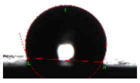	73.3±5.4	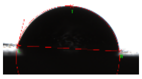
